# Impact of Coping Strategies on Health-Related Quality of Life in Young Adults with Multiple Sclerosis

**DOI:** 10.3390/brainsci14090866

**Published:** 2024-08-28

**Authors:** Viviana Lo Buono, Francesco Corallo, Edoardo Sessa, Giangaetano D’Aleo, Carmela Rifici, Angelo Quartarone, Lilla Bonanno

**Affiliations:** IRCCS Centro Neurolesi Bonino Pulejo, 98124 Messina, Italy; francesco.corallo@irccsme.it (F.C.);

**Keywords:** coping strategies, multiple sclerosis, health-related quality of life, rehabilitation

## Abstract

Multiple sclerosis (MS) is a chronic and progressive neurological disease that affects the central nervous system, resulting in a wide spectrum of cognitive, emotional, and physical deficits. The progressive course of MS poses significant challenges to patients and has a profound impact on health-related quality of life (HRQoL). The style of coping adopted plays a critical role in determining how individuals with MS adapt to and face the challenges of the disease and their overall well-being. This paper aims to examine the impact of coping strategies on HRQoL in young adults un-/minimally impaired (<5 years, EDSS ≤ 2.5) by MS (age 18–35 years). This retrospective cross-sectional cohort study included 98 young adults (33 males and 65 females) with relapsing–remitting MS who underwent neurological assessment using the Expanded Disability Status Scale. Participants completed the Italian version of the Multiple Sclerosis QoL-54 (MSQoL-54), which provides a physical and mental health score, and the Coping Orientation to Problems Experienced Inventory (Brief-COPE). The results showed a significant relationship between COPE scores and physical and mental health. Subjects affected by MS who tend to use more frequent coping strategies such as active planning, personal growth, and acceptance showed a better overall well-being and quality of life. These findings are relevant to clinical practice given the need to understand the coping variable to improve HRQoL. Understanding these relationships is crucial for developing effective interventions to enhance the well-being of MS subjects.

## 1. Introduction

Multiple sclerosis (MS) is the most commonly acquired demyelinating disorder of the central nervous system (CNS) [1]. The mean age of diagnosis is approximately 20–30 years, a critical period when individuals are planning their futures [2].

Prevalence has increased during the previous 50 years in both North America and Europe, with women experiencing a high incidence [3]. Relapsing–remitting MS (RRMS), primary progressive MS (PPMS), secondary progressive MS (SPMS), and progressive–relapsing MS (PRMS) are among the several phenotypes of MS that are categorized based on the patterns of progression of cognitive or physical impairment.

The most prevalent one is RRMS, which is characterized by distinct relapses where new symptoms appear or existing ones worsen [4].

The pathogenesis of MS is complex and involves a combination of genetic, environmental, and immunological factors. Clinical manifestations of disease occur when the immune system mistakenly attacks the myelin sheath that protects nerve fibers, leading to inflammation and nerve damage. This results in various symptoms, including fatigue, weakness, numbness, balance and coordination problems, and cognitive impairment, such as memory, attention, information-processing, and problem-solving difficulties. In addition, neuropsychiatric complications, such as anxiety and depression, can be caused by demyelination and inflammation as well as by the psychological impact of trying to adjust to an uncertain illness [5].

MS can significantly impact quality of life (QoL) due to its unpredictable nature and diverse range of symptoms that interfere with daily activities, mobility, and overall functioning, affecting a person’s independence and ability to participate in work, social, and recreational activities.

QoL is a generic term used to refer to an individual’s total well-being. In accord with the World Health Organization [6], QoL is influenced by physical and mental well-being, degree of independence, social relationships, personal views, and interactions with their surroundings. Various aspects of general well-being are influenced by health and how health, in turn, impacts overall life satisfaction. Health-related quality of life (HRQoL), specifically, assesses the potential long-term effects of an illness, disability, or disorder on an individual’s well-being [7].

An important psychosocial factor that influences HRQoL in neurodegenerative disorders is the coping style used by people to manage the disease state. Coping strategies are behavioral and cognitive modalities used to lessen the negative effects of stressful events [8]. Coping styles can be distinguished as follows: (a) emotion-focused coping, which refers to the capacity to control negative emotions; (b) problem-focused coping, which refers to strategies and actions aimed at mitigating the negative impact of the situation through an external change; (c) active coping, which targets the stressor directly; (d) avoidant coping, which is the act of escaping from emotional and cognitive experiences; (e) accommodative coping, which is directly related to a change in the personal goal standards due to perceived deficits; and (f) assimilative coping, which refers to proactive efforts to change unfavorable living circumstances and environmental limitations based on individual preferences [9]. Coping can be classified as either dysfunctional (increasing stress) or functional (adaptation), depending on how well or poorly this process goes.

In the past several years, there has been an increasing interest in the role that coping plays in MS outcomes, as it has been shown to be critical in helping people adjust to chronic illnesses.

The studies conducted in this field have demonstrated that MS subjects tend to employ dysfunctional avoidance or emotion-focused coping mechanisms, which are less effective in overcoming disease-related challenges. Conversely, MS subjects who use adaptive coping strategies report a better HRQoL [10]. In the study by Pakenham [11], acceptance and positive reframing were linked to better psychological well-being and life satisfaction in people with MS.

HRQoL measures are increasingly essential for clinical effectiveness and quality-of-care research, especially for chronic diseases. In this study, we analyzed the effects of several coping mechanisms on HRQoL in young adults with RRMS. Understanding these relationships is imperative to designing effective interventions to enhance the long-term well-being of people with MS.

## 2. Materials and Methods

### 2.1. Study Design

This retrospective cross-sectional cohort study included subjects with MS attending the outpatient clinic of Neurology at the IRCCS Centro Neurolesi “Bonino Pulejo” of Messina between April and June 2023. All participants provided informed consent in accordance with the Declaration of Helsinki.

### 2.2. Study Population

A total of 98 (33 males and 65 females) young adult subjects with RRMS were enrolled. All subjects had an average age of 28.68 years with a standard deviation of 6.97, and an average education level of 6.42 years with a standard deviation of 2.53. Demographic and clinical characteristics are shown in Table 1.

An experienced neurologist diagnosed multiple sclerosis (MS) based on the 2017 revised McDonald criteria during the examination and assessed the patients’ disability using the Expanded Disability Status Scale [12].

Inclusion criteria were the following: diagnosis of RRMS based on clinical history and MRI findings; no evidence of progressive disease on MRI or clinical examination for at least one year prior to enrollment; age between 18 and 35 years old; RR disease course; Expanded Disability Status Scale (EDSS) ≤2.5; Montreal Cognitive Assessment (MoCA) score ≤25; participants must provide written informed consent before participating in the research agreeing to all study procedures.

Exclusion criteria were the following: history of major psychiatric disorders; other neurological disorders; pathologies in comorbidity; use of antidepressant or psychotropic medications that could potentially influence cognitive functioning.

### 2.3. Outcome Measures

All MS subjects underwent a neuropsychological assessment. A battery of tests was administered to each patient by two trained neuropsychologists. Quality of life was assessed using the Italian version of the Multiple Sclerosis QoL-54 (MSQoL-54) [13]. The MSQoL-54 is a comprehensive HRQoL measurement system that incorporates both generic and MS-specific items into a single instrument [13]. This 54-item instrument generates 12 subscales: Physical Function (PF), Role Limitations Physical (RLP), Role Limitations Emotional (RLE), Pain (P), Emotional Well-Being (EWB), Energy (E), Health Perceptions (HP), Social Function (SF), Cognitive function (CF), Health Distress (HD), Overall Quality of Life (OQoL), and Sexual Function (Sex F). The summary scores provide two composite scores: physical health and mental health scores.

The coping strategies were assessed using the Coping Orientation to Problems Experienced Inventory (Brief-COPE) [14]. The Brief-COPE is a 28-item self-report questionnaire designed to measure effective and ineffective ways to cope with a stressful life event. The following subscales determine the coping styles used by the subject: Self-Distraction (SD), Denial (D), Substance Use (SU), Behavioral Disengagement (BD), Emotional Support (ES), Venting (V), Humor (H), Acceptance (A), Self-Blame (SB), Religion (R), Active Coping (AC), Use of Instrumental Support (UIS), Positive Reframing (PR), and Planning (P).

### 2.4. Statistical Analysis

A description of the groups was reported for demographic and clinical variables. Continuous variables were expressed as mean ± standard deviation, whereas categorical variables in frequencies and percentages. No parametric analysis was carried out because the results of the Shapiro normality test indicated that most of the target variables were not normally distributed. Spearman’s rank correlation was utilized to investigate potential relationships between COPE and physical and mental health scores. Analyses were conducted using an open source R4.2.2 software package. A 95% confidence level was set with a 5% alpha error. Statistical significance was set at *p* < 0.05.

## 3. Results

### 3.1. Correlation between COPE and Physical Health

Spearman correlation analysis revealed a significant relationship between COPE and physical health, a significant negative correlation between AC and HP (r = −0.21; *p* = 0.04), and a significant positive correlation between AC and PF (r = 0.29; *p* = 0.004), E (r = 0.21; *p* = 0.04), RLP (r = 0.27; *p* = 0.007), SF (r = 0.23; *p* = 0.02), and HD (r = 0.20; *p* = 0.04).

D correlates negatively with PF (r = −0.2; *p* = 0.04), but positively with HP (r = 0.28; *p* = 0.005).

We highlighted a negative significance correlation between UIS and PF (r = −0.21; *p* = 0.04), E (r = −0.27; *p* = 0.007), and HD (r = −0.31; *p* = 0.002), and between BH and RLP (r = −0.29; *p* = 0.004) and SF (r = −0.28; *p* = 0.005), and a positive significance correlation between BH and PF (r = 1; *p* < 0.001).

V correlates positively with HP (r = 0.23; *p* = 0.02) and negatively with HD (r = −0.30; *p* = 0.003). PR shows a significant positive correlation with E (r = 0.23; *p* = 0.02), RLP (r = 0.3; *p* = 0.002), SF (r = 0.27; *p* = 0.008), and HD (r = 0.34; *p* = 0.0006). P correlates positively with PF (r = 0.33; *p* = 0.0009), RLP (r = 0.28; *p* = 0.005), and SF (r = 0.21; *p* = 0.04).

Finally, we found a significant correlation between A and all items of physical health except for P (r = 0.14; *p* = 0.15); in particular, there was a significant negative correlation between A and HP (r = −0.34; *p* = 0.0006), and a significant positive correlation between A and PF (r = 0.34; *p* = 0.0005), E (r = 0.28; *p* = 0.005), RLP (r = 0.29; *p* = 0.003), SF (r = 0.22; *p* = 0.03), Sex F (r = 0.26; *p* = 0.009), and HD (r = 0.34; *p* = 0.0007).

### 3.2. Correlation between COPE and Mental Health

An intra-group analysis also showed a significant correlation between COPE and mental health.

AC showed a positive correlation with CF (r = −0.26; *p* = 0.01). We highlighted a significant negative correlation between UIS and HD (r = −0.34; *p* < 0.001) and EWB (r = −0.24; *p* = 0.01). BH only correlates negatively with OQoL (r = −0.21; *p* = 0.04). V correlates negatively with HD (r = −0.30; *p* = 0.002) and OQoL (r = −0.20; *p* = 0.04). PR shows a significant positive correlation with HD (r = 0.34; *p* < 0.001), EWB (r = 0.25; *p* = 0.01), and CF (r = 0.31; *p* = 0.002). We only found a significant positive correlation between P and CF (r = 0.21; *p* = 0.03) and between A and HD (r = 0.32; *p* = 0.001), OQoL (r = 0.26; *p* = 0.01), and CF (r = 0.28; *p* = 0.005). Finally, we highlighted a trend of significance between H and EWB (r = 0.19; *p* = 0.06), and SB and CF (r = 0.18; *p* = 0.07).

## 4. Discussion

Coping strategies play a crucial role in determining the HRQoL of individuals with MS by influencing symptom management in daily life. Young adulthood is a period of newfound independence and exploration, as individuals embark on new careers, passions, and relationships. However, this time frame is when MS is most likely to occur. The different coping strategies employed by young adults with MS can differentially affect both physical and mental health and influence the long-term management of one’s disease.

Our findings are mostly consistent with previous reports [15], demonstrating that active coping strategies for problems in young adulthood tend to have positive associations with physical health. A good capacity for venting was positively correlated with health perception, indicating that expressing emotions may contribute to a more positive perception of one’s health. In addition, positive reinterpretation, personal growth, and planning exhibited positive correlations with various aspects of physical health, indicating that finding meaning in adversity and planning may promote better physical well-being.

In our sample, acceptance of disease emerged as a particularly important coping strategy for enhancing overall physical health, with the exception of the pain dimension. Acceptance helps individuals adapt to the chronic nature of MS, reducing stress and improving overall well-being. It can lead to better management of symptoms and adherence to treatment regimens, thus positively affecting overall physical health. Pain in MS is a multifaceted symptom that may not be as readily influenced by acceptance alone. Pain perception is influenced by various factors, including neurological damage, psychological state, and individual pain thresholds. While acceptance can help reduce the emotional distress associated with pain, it might not significantly alter the physical sensation of pain. The neurobiological underpinnings of pain in MS involve demyelination and nerve damage, which are not directly impacted by coping strategies. Therefore, even if a person accepts their condition, the physiological processes causing pain remain unaffected. This is why acceptance might not show the same positive association with the pain dimension as it does with other aspects of physical health [16].

While this research focused on understanding and promoting effective pain management, studies have consistently identified pain acceptance as a strong correlator of positive physical and psychosocial adaptation to chronic pain [17]. In contrast with acceptance, denial of the problem or repression of one’s emotional state seem to lead to decreased physical functioning, such as avoidance-based coping strategies like behavioral disengagement, which may have detrimental effects on physical health.

Regarding mental health, active coping and coping flexibility, that is, engaging actively in the management of stressors, seem to enhance one’s ability to adapt and cope effectively to MS. Positive reinterpretation, planning, and acceptance tend to have positive associations with mental well-being, while avoidance-based coping strategies and relying solely on emotional support may have detrimental effects. Additionally, the ability to adapt and find humor in difficult situations may also contribute to better mental health outcomes.

Proactive health behaviors and problem-focused coping strategies like learning about symptom management appear to increase HRQoL and improve symptom control.

Providing young adults with information about coping strategies and their importance in managing stress and promoting well-being represents an important health outcome and, as well as educating individuals about the relationship between coping skills and HRQoL, can improve therapeutic adherence [18].

Healthcare providers should focus on promoting adaptive coping strategies in MS patients through comprehensive care plans that include psychological support, patient education, and empowerment programs. Interventions like cognitive behavioral therapy or mindfulness-based stress reduction can be particularly effective in teaching adaptive coping mechanisms.

The rehabilitation of coping skills is a crucial aspect of comprehensive care for individuals facing health challenges such as MS and it requires a multidisciplinary approach to empower patients to effectively manage stress, cope with daily challenges, and enhance overall well-being [19].

While physical disability is typically the focus of clinical assessments of MS patients, monitoring HRQoL is becoming more and more important.

MS presents unique challenges for young adults, striking during a crucial period of personal and professional development. The symptoms of MS, particularly fatigue and cognitive changes, can significantly hinder their ability to work, study, and maintain a normal lifestyle. These disruptions can lead to feelings of frustration, anxiety, and uncertainty about the future.

Given these challenges, it is essential to equip young adults with effective coping strategies. Through education and rehabilitation, they can learn to manage their symptoms, maintain their mental health, and continue to pursue their goals. Educational programs can provide proactive resources and knowledge, while therapeutic interventions and support systems can offer personalized guidance and emotional support.

Developing strong coping mechanisms, such as problem-solving skills, emotional regulation, mindfulness, and maintaining a healthy lifestyle, can empower young adults with MS to navigate their condition more effectively. By fostering resilience and adaptability, they can enhance their quality of life and remain engaged in their personal and professional pursuits despite the challenges posed by the disease.

This study has several potential limitations that may affect the validity and generalizability of its findings. One of the main limitations is that it is a retrospective analysis, so longitudinal design can provide valuable insights into changes in coping mechanisms over time and their relationship with disease progression.

It would also be interesting to extend this research to subjects recently diagnosed with other forms of MS or experiencing stronger disability to contribute to a more comprehensive understanding of coping mechanisms. Studying participants with varying levels of disability could allow the exploration of how coping mechanisms evolve as the disease progresses and functional limitations increase.

Another important limitation of this research is its small sample size, which precluded the generalization of clinical results and prevented the allocation of sufficient sample sizes to each subgroup for meaningful comparisons of gender differences in the use of different coping strategies.

Future studies should employ larger sample sizes to mitigate the risk of low statistical power contributing to non-significant findings. Additionally, future research could investigate the efficacy of psychotherapeutic or educational interventions aimed at enhancing empowerment and active coping strategies, and, subsequently, the health outcomes of MS subjects.

## 5. Conclusions

Coping strategies significantly influence the HRQoL of young adults with RRMS. Adaptive coping mechanisms, such as active coping, acceptance, positive reframing, and planning, correlate with improved physical and mental health outcomes. Conversely, maladaptive strategies like denial and behavioral disengagement are linked to a poorer HRQoL. Given the substantial impact of MS on young adults’ lives, the development of effective coping skills is essential for managing disease-related challenges. The routine assessment of coping strategies should be integrated into clinical care for individuals with MS. A holistic approach prioritizing subjective well-being is crucial for clinicians to optimize health outcomes.

## Figures and Tables

**Table 1:** Demographic and clinical characteristics of MS subjects.

Neuropsychological Test	Median (I–III Quartile)
COPE	
Self-Distraction	6.0 (4.0–6.75)
Active Coping	7.0 (6.0–8.0)
Denial	3.0 (2.0–5.0)
Substance Use	2.0 (2.0–2.0)
Use of Instrumental Support	4.5 (4.0–6.0)
Emotional Support	4.0 (4.0–6.0)
Behavioral Disengagement	4.0 (2.0–5.0)
Venting	4.0 (3.0–6.0)
Positive Reframing	6.0 (4.5–7.0)
Planning	6,5 (5.0–8.0)
Humor	4.0 (3.0–6.0)
Acceptance	7.0 (6.0–8.0)
Religion	5.0 (4.0–7.0)
Self-Blame	5.0 (4.0–7.0)
PHYSICAL HEALTH	
Physical Function	11.05 (7.65–15.51)
Health Perception	8.5 (6.76–10.2)
Energy	5.76 (4.8–7.2)
Role Limitations Physical	6.0 (0.0–12.0)
Pain	7.7 (5.12–10.01)
Sexual Function	7.33 (4.66–8.0)
Social Function	9.0 (6.99–10.99)
Health Distress	8.25 (6.05–9.35)
TOTAL	63.88 (52.22–73.68)
MENTAL HEALTH	
Health Distress	10.5 (7.7–11.9)
Overall Quality of Life	11.4 (9.0–13.79)
Emotional Well-Being	16.24 (12.76–20.88)
Role Limitations Emotional	15.98 (0.0–24.0)
Cognitive Function	9.75 (7.31–12.0)
TOTAL	62.49 (44.02–79.93)

## Data Availability

The data that support the findings of this study are available on request from the corresponding author due to reasons of sensitivity.
